# Body composition, dual-energy X-ray absorptiometry and obesity: the paradigm of fat (re)distribution

**DOI:** 10.1259/bjrcr.20170078

**Published:** 2019-03-14

**Authors:** Federico Ponti, Andrea Plazzi, Giuseppe Guglielmi, Giulio Marchesini, Alberto Bazzocchi

**Affiliations:** 1 Diagnostic and Interventional Radiology, IRCCS Istituto Ortopedico Rizzoli, Bologna, Italy; 2 Unit of Metabolic Diseases & Clinical Dietetics, University of Bologna. Sant'Orsola - Malpighi Hospital, Via G. Massarenti 9, Bologna, Italy; 3 Department of Radiology, University of Foggia, Viale Luigi Pinto 1, Foggia, Italy; 4 Department of Radiology, Scientific Institute “Casa Sollievo della Sofferenza” Hospital, Viale Cappuccini 1, San Giovanni Rotondo, Foggia, Italy

## Abstract

**Objective:**

The amount of lean and fat tissues in different body compartments is likely to drive the cardiovascular risk. The longitudinal effects of changes of lean and fat mass, particularly following weight loss programs, cannot be reliably identified by the sole measurement of anthropometry. We discuss this problem on the basis of data collected in obese females with the use of dual-energy X-ray absorptiometry (DXA), anthropometry and laboratory.

**Methods:**

We present longitudinal data in six obese females (three pairs—weight stable, weight loss, weight increase) assigned to a medical treatment. All patients underwent whole-body scan (Lunar iDXA, GE Healthcare, WI) and laboratory analysis (blood fasting glucose, total low-density lipoprotein and high-density lipoprotein cholesterol, triglycerides) before treatment and after 12 months. Fat mass and non-bone lean mass were assessed at whole-body and regional levels. Android visceral adipose tissue was estimated by a recently validated software.

**Results:**

The most common anthropometric measures (body mass index, waist circumference) were totally ineffective in documenting the changes in body composition in 12 month follow-up, whereas DXA could detect regional changes, which were paralleled in part by changes in biochemical indices.

**Conclusion:**

Serial DXA measurements could provide a comprehensive assessment of body compartments, independent of changes in classic anthropometry (body mass index and waist circumference), identifying a significant redistribution of lean and fat mass and providing clues to explain changes in cardiovascular risk profile.

## Introduction

The World Health Organization (WHO) defines overweight and obesity as “abnormal or excessive fat accumulation to the extent that health may be impaired”.^1^ This condition represents a significant risk factor for comorbidities, from cardiovascular and respiratory diseases to metabolic alterations and cancer development.^2^ Diet, physical activity and behavior therapy are standard treatments for obesity, which may also require pharmacologic intervention and surgery.^3,4^

### Why body composition?

Fat distribution and/or ectopic fat accumulation (excessive triglyceride content in the liver and muscles, fat accumulation in the neck, as well as pericardial, perivascular fat, renal sinus, omental fat—*i.e.* any type of visceral fat) are reported to drive cardiometabolic and vascular risks more than obesity itself, with subcutaneous fat carrying a lower risk compared with visceral fat. In the presence of positive energy balance, the inadequate expansion of subcutaneous adipose tissue (SAT) is a cause of lipid overflow into the visceral depots as well as into non-adipose tissues (namely, the hepatic parenchyma, islet cells, exocrine pancreas), a phenomenon called lipotoxicity, which promotes cardiometabolic consequences.^5^

The overall risk is also dependent on lean body mass (largely identified as muscle mass), because it also participates in metabolic function. Lean mass is the compartment responsible for energy expenditure and regulates substrate metabolism. A relative decrease of lean mass, the so called “sarcopenic obesity” (SO), characterized by an excess of body fat and reduced muscle mass and/or strength significantly contributes to health risks.^6,7^

### Why dual energy X-ray absorptiometry (DXA)?

The body mass index (BMI) remains the most widely used marker of obesity in epidemiological studies, but may be largely inadequate for the assessment of the metabolic status,^8^ particularly following weight loss programs, when the benefits of weight loss may be also related to regional changes in body fat and lean mass.^9,10^ Also waist circumference, thanks to easy measurement, is frequently used as surrogate for body changes, but it only discriminates between visceral and peripheral weight loss.

**Figure 1.**
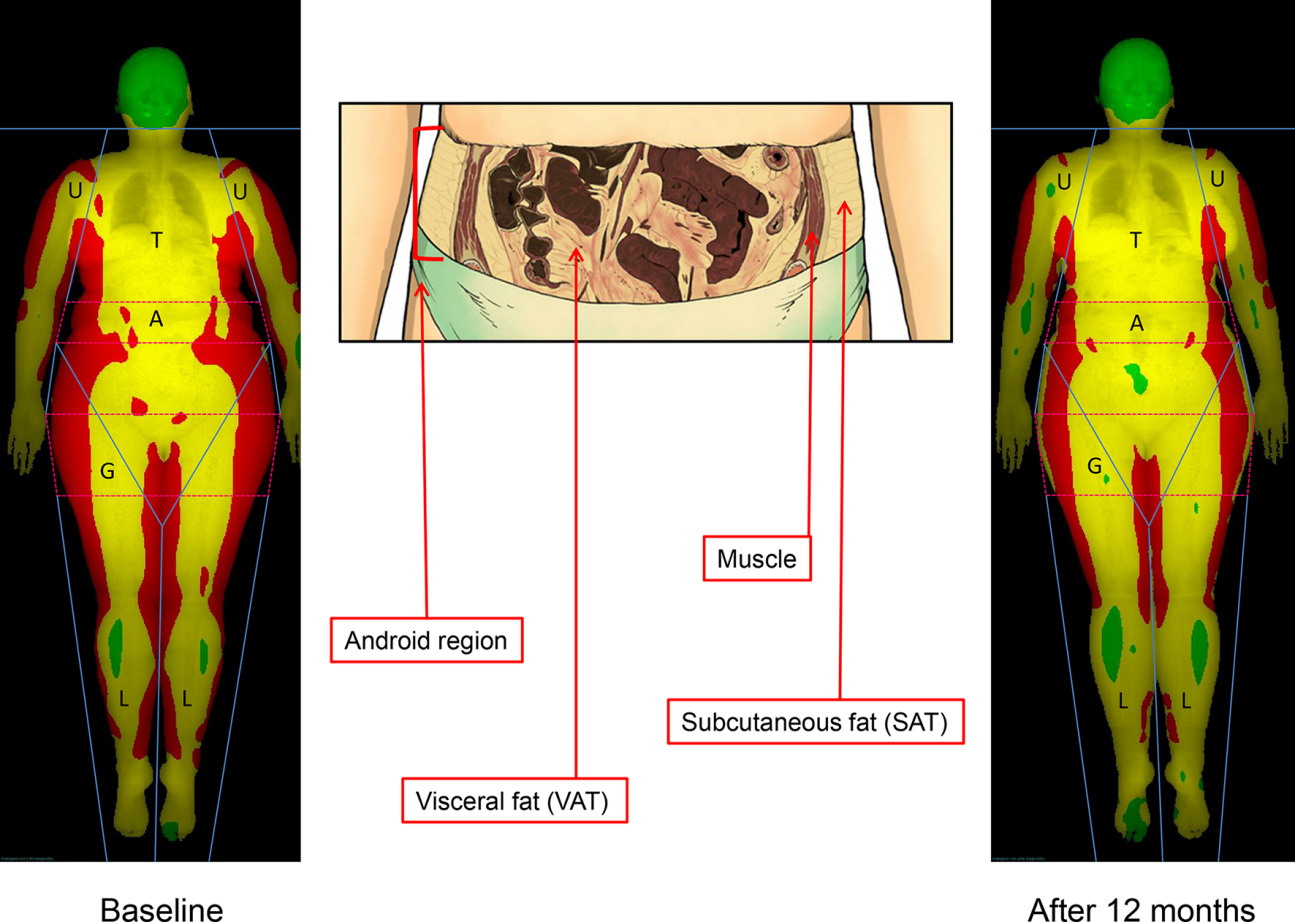
DXA body composition analysis. Colored soft tissues map of whole-body scan by DXA (red, high fat percentage—conventionally >60%; yellow, medium fat percentage—between 25 and 60%; green, low fat percentage—<25%) of the subject A3, at baseline (left) and after 12 months (right), in order to outline regional changes of body composition (trunk,T; upper limbs, U; lower limbs, L; android, A [the portion of the abdomen included between the line joining the two superior iliac crests and extending cranially up to the 20% of the distance between this line and the chin]; gynoid, G [the portion of legs stretching caudally from the femoral great trochanter to a distance double of the android region]). An enlargement of the android region is also presented (center) with the representation of the abdominal muscles and subcutaneous and visceral fat compartments (anatomic picture by Andrea Plazzi, Imola, Italy). DXA, dual-energy X-ray absorptiometry; SAT, subcutaneous adiposetissue; VAT, visceral adipose tissue.

More accurate techniques (bioelectrical impedance analysis, imaging techniques) have gained popularity for a more precise assessment of the effects of treatment.^11^ In particular, DXA guarantees a precise assessment of the three main body components [(bone mineral content, non-bone lean mass (LM), and fat mass (FM)] both at whole body and at regional level, as well as the measurement of the amount of visceral adipose tissue (VAT), thanks to the most recent software developments.^12,13^ Because of its high accuracy and precision, low-cost, large availability, the DXA approach candidates as a standard technique to measure the longitudinal changes in body composition following the treatment of obesity or treatment failure (Figure 1).

## Methods

We retrospectively present longitudinal data collected in the period from February 2012 to December 2014 in six obese females come from a clinical population of our centre (three pairs—weight stable, weight loss, weight increase on the basis of BMI with different trend of body composition evaluated by DXA) . Subjects were assessed by obesity specialists and assigned to either an intensive 3 month cognitive behavioral treatment (CBT) program or a 1 month nutritional counseling program (NCP) on the basis of initial BMI and psychological profile. In particular subjects A1, B1, A3 and B3 were enrolled in a CBT program. CBT is a psycho-educational, cognitive-behaviorally oriented process, aimed at building a personal system of self-control capable of dealing appropriately with stimuli that induce to uncontrolled feeding and with change in lifestyle for weight control. CBT provides patients with the principles and techniques for the modification of their eating habits and activities. It combines a strong educational component (self-monitoring, goal-setting, stimulus-control, alternative behaviors, problem-solving, cognitive restructuring) with specific recommendations related to diet and exercise.

Treatment consisted of 13 group-sessions (2 h, in groups of 12–15 patients), on a weekly basis, guided by a multidisciplinary team of professionals, consisting of a medical doctor, nutritionist and psychologist, with a predetermined work program based on a guided self-help manual.

Subjects A2 and B2 were addressed to a pure educational approach, aimed at providing patients with practical information on healthy diet and on how to reduce calorie intake or increase energy expenditure by habitual physical activity (NCP). In our setting, NCP consisted of five meetings (2 h, on a weekly basis, in groups of 20–25 patients) chaired by an expert (nutritionist/dietitian) that explained the principles of healthy diet (food portions defined by the WHO, the food pyramid and the Mediterranean diet), and healthy lifestyles (reduction in caloric intake and increasing physical activity in order to obtain a slow, but progressive weight reduction).

For each patient anthropometric measures (BMI and waist circumference), DXA parameters and biochemical indices (blood fasting glucose, total, low-density lipoprotein and high-density lipoprotein cholesterol, triglycerides) were evaluated at the beginning of treatment and after 12 months (Table 1).

**Table 1.** Longitudinal changes in body composition in obese females

Parameter (baseline and % change)	Weight loss		Weight maintenance		Weight gain	
	A1	B1	A2	B2	A3	B3
Age (years)	57	65	59	69	63	70
**Anthropometric parameters**						
Baseline BMI (kg/m^2^)	37.5	31.1	37.8	32.5	45.6	48.5
Baseline waist circumference (cm)	116	86	97	94	144	148
Δ Weight (kg)	−7.8	−2.4	−0.3	−0.1	+7.1	+3.3
Δ Body mass index (%)	−7.8	−3.0	−0.2	−0.2	+4.4	+2.5
Δ Waist circumference (cm)	−7	−2	−2	+3	−2	+2
**DXA parameters**						
Δ Total fat mass (%)	−**17.2**	−**3.3**	−**2.5**	**+3.1**	**+3.3**	**+4.2**
Δ Total lean mass (%)	**+2.9**	−**2.7**	+1.8	−**3.3**	**+5.8**	+0.4
Δ Android fat mass (%)	−**22.1**	+4.2	−**6.0**	**+9.3**	+1.4	**+14.0**
Δ Android lean mass (%)	−0.5	+2.1	−**9.4**	−**6.8**	+2.8	**+11.6**
Δ Gynoid fat mass (%)	−**16.8**	+0.2	−**9.3**	−1.9	**+12.0**	−**5.7**
Δ Gynoid lean mass (%)	+2.6	+3.5	+1.0	−2.5	+1.2	−1.2
Δ Visceral adipose tissue (%)	−**28.9**	**+13.4**	−**13.7**	**+12.6**	−**24.1**	**+12.4**
ΔSubcutaneous adipose tissue (%)	−**18.1**	+1.6	−3.7	+4.5	**+56.2**	**+15.0**
ΔTrunk Fat Mass (%)	−**31.3**	+0.9	−1.5	**+6.4**	**+6.7**	**+7.2**
ΔTrunk Lean Mass (%)	−**11.2**	+2.5	+2.0	−**5.4**	**+11.4**	+1.9
ΔLeg Fat Mass (%)	**+3.4**	**+6.0**	−**3.8**	−1.1	−1.8	−0.3
ΔLeg Lean Mass (%)	**+25.1**	+3.1	−1.2	−1.2	+1.8	−1.9
ΔArm Fat Mass (%)	−**10.5**	+1.8	−3.9	−0.3	−**6.1**	−1.3
ΔArm Lean Mass (%)	**+7.0**	+1.1	−0.9	−**6.2**	−3.4	+2.3
Baseline Skeletal Muscle Index (%)(n.v.: M,>30; F,>25)^14^	18.4	20.2	24.1	24.4	24.4	19.6
ΔSkeletal Muscle Index (%)	**+30.8**	+0.4	−0.9	−2.3	−3.8	−3.3

BMI, body mass index.

The values highlighted in bold type are significant because the differences are in excess of the Least Significant Change, calculated as % coefficient of variation x 2.77 for DXA parameters, on the basis of the precision in our center.

DXA measurements were made using a total-body scanner (Lunar iDXA 2005, Madison, WI; enCORE^TM^ 2011, software v. 13.6). The iDXA is a narrow fan-beam DXA instrument with a high weight limit (up to 204 kg) and a relatively wide scanning space (66 cm) designed to accommodate obese subjects.

At the end of the scan, a total body and regional analysis is automatically made by the software, which provides measures of different parameters. FM, non-bone LM were considered for final analysis on the whole-body, android region (a portion of the abdomen included between the line joining the two superior iliac crests and extending cranially up to the 20% of the distance between this line and the chin), gynoid region (a portion of legs stretching caudally from the femoral great trochanter to a distance double of the android region), trunk, upper limb and lower limb.

Visceral fat analysis was performed by CoreScan^TM^, a software option for the assessment of visceral fat (mass in g, and volume in cm^3^) in the android region. The detection of the layer thickness of the SAT at sides of the android region allowed the software to map total SAT compartment. The amount of VAT was derived by subtracting SAT from the total android FM.

We reported information on the reproducibility of DXA (least significant change—LSC) provided by the DXA machine. LSC is the least amount of parameter change that can be considered statistically significant. The International Society of Clinical Densitometry recommends calculating this for a 95% confidence level, which is done by multiplying the precision error (coefficient of variation or root mean square standard deviation in absolute terms) by 2.77. Knowledge of precision is particularly important since the physiological changes in tissue, or the changes induced by therapy, are very small. Thus to decide if a change is significant (*i.e.* clinically meaningful), it is important to know the magnitude of the inherent variation in measurement. If the change equals or exceeds the LSC, then this can be considered to be a genuine biological change.

The scope of this case report is to provide some hints regarding a problem we are facing in our everyday practice, and we decided to pinpoint the problem of the scarce correlation between BMI and body fat (or body fat distributions) by a few examples.

Each subject signed an informed consent prior to participation. The study was conducted according to the Declaration of Helsinki and was approved by the local ethical committee.

## Results

### Weight loss

Both subjects A1 and B1 were enrolled in a CBT program.^15^ In Subject A1, the reduced body weight and BMI (−7.8%) were paralleled along the year by a healthy redistribution of body compartments at DXA analysis, with markedly decreased VAT and a systematic increase in lean mass in the different districts. Laboratory data were consistent with reduced cardiovascular risk showing reduced levels of total (from 216 to 163 mgdl^−1^) and low-density lipoprotein cholesterol (from 133 to 87 mgdl^−1^), as well as lower triglycerides (from 84 to 72 mgdl^−1^).

In Subject B1, a moderate decrease in body weight and BMI was accompanied by negative changes in body composition. Android and visceral fat mass increased, in association with a remarkable loss of total lean mass. No changes in laboratory values were observed despite weight loss.

### Weight maintenance

In Case A2, a positive redistribution of lean and fat mass was observed in body compartments at 12 months, following patient’s attendance to a nutritional counseling program, without any significant change in weight and BMI; laboratory data remained stable after 1 year.^15^

The weight of Subject B2, who attended the same nutritional counseling program, remained also stable (−0.19 %), whereas his body composition was characterized by a progressive increase in VAT.

### Weight gain

Patient A3 was also enrolled into a CBT program of patients A1 and B1. During the 12 month follow-up, body weight increased; however, DXA demonstrated a remarkable redistribution of body composition parameters. In the presence of a modest gain in total fat mass (TFM) and in SAT, VAT decreased by nearly 25%. On the contrary, lean mass variably increased in the various districts. Laboratory data confirmed a reduced cardiometabolic risk, and a systematic reduction in triglyceride (from 150 mg dl^−1^ to 132 mgdl^−1^) and glucose levels (from 103 mg dl^−1^ to 93 mgdl^−1^).

At 1 year follow-up, Patient B3, enrolled into the same behavioral program, experienced a modest increase in weight and BMI, accompanied by very negative changes in body composition. Fat depots increased systematically, without any significant change in lean mass (total lean mass (TLM), +0.4%).

## Discussion

It is known that serial DXA measurements provide a comprehensive assessment of body compartments, independent of changes in classic anthropometry (BMI and waist circumference).^14,16–18^

The report suggests that DXA parameters might be used to modify the clinical approach or to explain changes in clinical parameters, unexpected on the basis of the classical anthropometric approach.

Both subjects A1 and B1 experimented a reduction of body weight and BMI, but with an opposite body composition’s trend (decrease and increased of VAT, respectively). As the matter of fact the diet history of Patient B1 revealed that weight loss was accomplished only by severe calorie restriction, without any increase in habitual physical activity, at the contrary of the Patient A1.

The body weight of subjects A2 and B2 remained stable despite to redistribution of body composition, positive in case of A2 who was totally compliant to the suggested changes in dietary intake and habitual physical activity (he changed food preferences and started a minimum of leisure-time physical activity), in opposition to B2.

The cardiovascular risk profile of Subject B2 could be maintained at baseline values only by increased statin treatment.

An explicative example is patient A3 *vs* B3, where the most important difference is the finding that increased BMI could be observed in the presence of increased visceral fat (B3), but also in the presence of decreased visceral fat (A3). Notably, laboratory values tend to move in opposite directions, with a trend towards decreased blood glucose and lipid only in the presence of decreased visceral fat.

At follow-up, Patient A3 reported he had started an intense physical activity program, complying with the targets suggested in the course of the cognitive program, without any significant reduction of food intake.

On the contrary, Patient B3 was totally non-compliant to the same behavioral program. He did not change his lifestyle, both on diet and on physical activity side, so an intensification of pharmacologic treatment was needed to maintain his glucose and lipid profile within entry values.

This case review shows simplified picture of the potential role and possibilities of DXA in the management of obese patient, through few selected obese patients of clinical practice, when is depicted the different body composition trend measured by DXA over 12 months despite and opposite or stable trend of BMI.

## Conclusion

Today, DXA has reached a new important frontier in the analysis of body composition, bridging the historical gap of visceral fat assessment, with unexplored clinical implications.

Combining changes in individual body compartments by DXA with their related clinical and functional aspects (*e.g.* endocrine, metabolic and inflammatory profiles) is likely to provide a useful piece of information for the identification of ‘metabolically healthy body composition’^19^ and sarcopenic obesity,^20^ especially during weight loss strategies.^21^

Obviously, much larger studies are needed to support this anecdotal evidence or narrative medicine.

## Learning points

1. The cases presented illustrate the modification of multiple body composition parameters assessed by dual-energy X-ray absorptiometry (DXA), one of the most accurate and suitable techniques in today clinical practice and research,
2. On the basis of data collected in six females (three pairs—weight stable, weight loss, weight increase -), underwent to medical treatment for obesity, the longitudinal effects of changes of lean and fat mass compartments in a 12 month follow-up cannot be reliably identified by the sole measurement of anthropometry.
3. The use of DXA identified a significant redistribution of lean and fat mass, providing clues to explain changes in cardiovascular risk profile.
4. The analysis of body composition by DXA may provide very important information for clinicians with a potential impact of DXA equipments in the management of the disease and, in particular, in the prevention of its complications.
